# Cost-effectiveness of empagliflozin for the treatment of heart failure with reduced ejection fraction in China

**DOI:** 10.3389/fcvm.2022.1022020

**Published:** 2022-11-16

**Authors:** Haiqiang Sang, Yiming Wan, Zhenzhou Ma, Shengye Zhang, Qiuping Zhao

**Affiliations:** ^1^Department of Cardiology, The First Affiliated Hospital of Zhengzhou University, Zhengzhou, China; ^2^Department of Cardiology, Fuwai Central China Cardiovascular Hospital, Zhengzhou, China

**Keywords:** cost-benefit analysis, empagliflozin, heart failure with reduced ejection fraction, pharmacoeconomics, medical decision-making

## Abstract

**Aim:**

To determine the pharmacoeconomics of empagliflozin for the treatment of heart failure (HF) with reduced ejection fraction in China and to provide evidence-based reference for clinical rational drug selection and medical decision-making.

**Research design and methods:**

We used the Markov model to evaluate the cost-effectiveness of empagliflozin for the treatment of HF with reduced ejection fraction (HFrEF). We evaluated the cost-effectiveness of the standard treatment in addition to empagliflozin (empagliflozin group) vs. the cost-effectiveness of the standard treatment alone (standard treatment group).

**Results:**

We found that each additional quality-adjusted life year (QALY) in the empagliflozin group costed $3,842.20 more, which was less than China’s gross domestic product (GDP) per capita in 2021 ($11,981). The steady-state mortality in the two groups was the key factor affecting the incremental cost-effectiveness ratio (ICER). Probabilistic sensitivity analysis revealed that when the willingness-to-pay (WTP) threshold was one time the GDP per capita in 2021 ($11,981) and three times the GDP per capita in 2021 ($35,943), the probability of the empagliflozin group being cost-effective was 85.8 and 91.6%, respectively.

**Conclusion:**

Compared with the standard treatment alone, the addition of empagliflozin to the standard treatment was more cost-effective for the treatment of HFrEF in China.

## Introduction

Heart failure (HF) is an important global public health issue. According to a survey, the global prevalence of HF is approximately 1.3%, and there are more HF patients in developing countries than in developed countries. In China, there are more than 8.9 million patients with HF (1–3). The annual treatment cost per patient with HF in 2014 was approximately $4,550.45 (4). The hospitalization cost accounted for approximately 66% of the total cost of HF treatment (5). Therefore, if the hospitalization rate for HF is reduced, the HF treatment cost and global disease burden will decrease.

HF is categorized into HF with reduced ejection fraction (HFrEF) and HF with preserved ejection fraction (HFpEF). Sodium-glucose linked transporter 2 inhibitors (SGLT2is) are used for the treatment of diabetes. However, in recent years, with the advancement in research, the latest guidelines and consensus on HF in many countries recommend the use of SGLT2i for the treatment of HFrEF (6, 7). Examples of SGLT2is include canagliflozin, dapagliflozin, and empagliflozin. China’s guidelines for the primary diagnosis and treatment of chronic HF (2019) recommend dapagliflozin for the treatment of HFrEF (8). Packer et al. (9, 10) conducted a study on the effects of empagliflozin in patients with HFrEF (EMPEROR-Reduced trial) and revealed that the addition of empagliflozin to the standard treatment regimen could improve outcomes in patients with HFrEF (with or without diabetes), and the risk of hospitalization for HF was significantly reduced. The findings of the study adds new evidence for the use of SGLT2is in patients with HFrEF.

SGLT2is were first developed as a novel drug for the treatment of type 2 diabetes mellitus, mainly by inhibiting glucose reabsorption by the proximal renal tubular SGLT protein family. However, the mechanism of the CV benefit of SGLT2is remains unclear. Some studies have proposed some possible mechanisms: it is generally believed that SGLT2is can exert cardiac benefit through sodium drainage, improving cardiac energy metabolism, producing anti-inflammatory effects, and reducing sympathetic hyperactivity (11–13). In addition, SGLT2is can reduce LV volume and reverse LV remodeling to a degree, which explains the improvement of LV systolic function by SGLT2is (14, 15).

In addition to the effectiveness of a treatment, its economic benefit is another important factor in medical decision-making. Cost-effectiveness analysis is an effective method to evaluate the value of drugs by quantitatively comparing the treatment cost and effectiveness of different treatment strategies (16). Presently, there are no pharmacoeconomic studies on the treatment of HFrEF with empagliflozin in China. Economic evaluation seeks to assess the cost-effectiveness of empagliflozin in comparison to the standard treatment for HErEF. The Markov model is used to study the state and state transition of a system, and it has been widely used in the simulation of long-term chronic diseases in pharmacoeconomic evaluations. In this study, we used the Markov model to analyze the cost-effectiveness of the regimen for the treatment of HFrEF and evaluated the economic benefits of combined treatment for HErEF in China to provide evidence-based information for clinical rational drug use and medical decision-making.

## Patients and methods

### Clinical data

In this study, the principles and methods of pharmacoeconomics were used to conduct a cost-benefit analysis of empagliflozin in the treatment of HFrEF in China, and to provide evidence-based reference for clinical rational drug selection and medical decision-making. We used the data of the EMPEROR-Reduced study (9). We randomly divided 3,730 patients with HFrEF from 20 countries (including 134 Chinese patients) into two groups: the empagliflozin group (*n* = 1,863) and standard treatment group (*n* = 1,867). All patients received the standard HF regimen (including diuretics, angiotensin converting enzyme inhibitors/angiotensin 2 receptor blockers, β blockers, angiotensin receptor enkephalin inhibitors, and salt corticosteroid receptor antagonists). In addition, patients in the empagliflozin group received 10 mg of empagliflozin once a day, while patients in the standard treatment group received a placebo.

### Rationale and structure of the model

**FIGURE 1:**
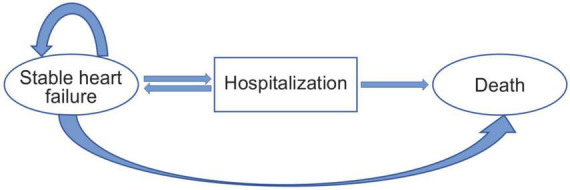
Patients occupying the health states shown in the ovals. Patients transitioning from different health states represented as arrows based on the transition probabilities.

In this study, the Markov model (designed using the Excel 2019 software) was used for the analysis and comparison of cost-effectiveness between the empagliflozin and standard treatment groups. Based on the EMPEROR-Reduced study, the following two independent basic states were established according to the development and prognosis of the disease: the stable HF and death states and transitional (hospitalization) state. According to the Chinese HF patient registration study (China-HF), the average age of 13,687 inpatients with HF from 132 hospitals across the country was 65 ± 15 years. Typically, most patients with HF are elderly. For diseases with high mortality, the shorter the cycle, the higher the accuracy of the results. Therefore, the simulation time limit of this model was set at 20 years, and the cycle was set to 1 month, with a total of 240 cycles. The average age of the selected patients in the EMPEROR-Reduced study was approximately 67 years; therefore, a life expectancy of 86 years was considered as the simulated termination age. The model used a half-cycle correction to prevent the overestimation of the expected survival time. At the same time, this study assumed that the population in the model received the same treatment, drug compliance was 100%, and type and doses of the drugs were unchanged within the entire model; the initial state of all the patients at the beginning of the model was stable, and the patients could only be in one state in the same cycle; the duration of hospitalization for the hospitalized patients was not more than 1 month. The state of death was absorptive, that is, once a patient was in the state of death, the patient could no longer transfer to other states. The Markov model of state transition for patients with HFrEF is shown in Figure 1. The model followed the standard structure of the HF model (17, 18).

### Markov model parameters

#### Transitional probability

In this study, according to the number of patients in each health state in the EMPEROR-Reduced study, the steady state mortality, hospitalization state mortality, and steady state hospitalization rates were calculated. With reference to previous studies (16), the transitional probability in this model was obtained using the transformation formula: *r=—[ln(1—P)]/T;p=1—exp[—rt]*, where r is the instantaneous incidence, P the incidence within the observation time limit, T the observation time limit, p the transfer probability of 1 cycle, and t the cycle. The monthly transitional probability between the health states of patients with HFrEF is shown in Table 1. In addition, during the simulation process of this model, the probability of non-cardiovascular (CV) death, that is, the basic probability of death, was included. According to the *China Health Statistics Yearbook: 2020*, the monthly probabilities of non- CV death for people aged 67–69, 70–74, 75–79, 80–84, and 85–86 years old were 0.00082, 0.00103, 0.00143, 0.00291, and 0.00822 (19), respectively. This is shown in Table 1.

**TABLE 1:** Input parameters of the Markov model.

Parameters	Value	Standard deviation	Range	Distribution	Reference	Note
**Transitional probability in the empagliflozin group**						
Stable heart failure to hospitalization	0.01449	0.00074	0.01304–0.01594	Beta	(9)	± 10% of the mean
Stable heart failure to CV death	0.00624	0.00032	0.00561–0.00686	Beta	(9)	± 10% of the mean
Hospitalization to CV death	0.03865	0.00197	0.03478–0.04251	Beta	(9)	± 10% of the mean
**Transitional probability in the standard group**						
Stable heart failure to hospitalization	0.02171	0.00111	0.01954–0.02389	Beta	(9)	± 10% of the mean
Stable heart failure to CV death	0.00657	0.00034	0.00592–0.00723	Beta	(9)	± 10% of the mean
Hospitalization to CV death	0.04392	0.00224	0.03953–0.04831	Beta	(9)	± 10% of the mean
**Transitional probability of non-CV mortality by age**						
67–69	0.00082				(19)	Local data
70–74	0.00103				(19)	Local data
75–79	0.00143				(19)	Local data
80–84	0.00291				(19)	Local data
85–86	0.00822				(19)	Local data
**Cost**						
Empagliflozin plus standard treatment	$55.07	$5.62	$44.06-$66.09	Gamma	*www.yaozh.com*	± 20% of the Mean
Standard treatment	$35.11	$3.58	$28.09-$42.13	Gamma	(5)	± 20% of the Mean
HF hospitalization	$1,408.13	$578.83	$974.20-$3,243.21	Gamma	(19)	Local data
Discounted rate	5%		1%-8%		(22)	
**Utility weight**						
Stable heart failure	0.871	0.088	0.783-0.959	Beta	(21)	95% CI
HF hospitalization	0.215	0.174	0.041-0.389	Beta	(21)	95% CI

CV, cardiovascular; HF, heart failure.

#### Cost, utilities, and discount

The cost in this model was the direct medical cost of the patients. The individual differences of the direct non-medical, hidden, and indirect costs are large and difficult to measure, and, therefore, were not taken into consideration. The direct medical cost of the patient included the monthly cost of empagliflozin, monthly cost of standard treatment, and cost of each hospitalization (including the fee for the treatment of various adverse events during hospitalization). The monthly standard treatment fee and cost of each hospitalization for HFrEF were derived from existing literature (5, 19). The unit price of empagliflozin was obtained from the latest winning price published at *www.yaozh.com* in China. This website is an early big data service provider that carries out in-depth processing of medical data, big data mining, achievement output, and empowerment in China. All fees were converted using the following exchange rate: 6.373 ¥/USD (The People’s Bank of China) (20). The utility value of each health state in this model was derived from reference (21), and quality-adjusted life years (QALYs) were used as the output index. In addition, the cost and health utility value discount rate of 5%, according to reference (22), was used in this study. The parameters of the Markov model and their distribution are shown in Table 1.

### Sensitivity analysis

In this study, Excel 2019 was used for sensitivity analysis to verify the robustness of the model’s simulation results. In the one-way sensitivity analysis of all parameters in the model, the influence of a parameter on the incremental cost-effectiveness ratio (ICER) robustness was verified using the value range of a parameter, while all other variables remained constant. The ranges of each hospitalization cost for HFrEF and health utility values were obtained from previous studies (19, 21). The range of the other costs and probability range of each transfer were 20% and 10% above and below the baseline value, respectively, and the discount rate was 1–8%. This is shown in Table 1.

This study conducted a scenario analysis of the cost of empagliflozin, each HFrEF hospitalization, and time horizon. According to the trend of centralized drug procurement in China, the cost of empagliflozin was reduced by 20, 40, and 60% to examine its impact on the ICER. For HFrEF hospitalization, various levels of hospitals had different hospitalization costs: incorporating town-level hospitals ($979.48), county-level hospitals ($1,138.66), municipal hospitals ($1,813.89), provincial hospitals ($1,863.57), and ministerial hospitals ($3,260.76) (19). The time horizon was adjusted to 10, 15, 20, and 25 years to explore its impact on the ICER.

The advantage of the probability sensitivity analysis was that the influence of multiple uncertain factors on the simulation results of the model could be considered at the same time. In this study, using the probability sensitivity analysis, we could predict the cost-effectiveness probability of the empagliflozin group under the different willingness-to-pay (WTP) levels. Through Monte Carlo random sampling simulation (1,000 iterations), referring to previous research (16), it was determined that the transitional probability and health utility value in this model conformed to the Beta distribution, and the cost conformed to the Gamma distribution.

## Results

### Model validation and clinical results

The average age of the simulated population in this study was 67 years. Our model predicted that all-cause mortality at 16 months in the empagliflozin group would be 11.5%, CV mortality 10%, and total hospitalization for HFrEF rate 23.2%. All-cause mortality at 16 months in the standard treatment group was 12.5%, CV mortality 10.5%, and hospitalization for HFrEF rate 34.5%. The results of the simulations were close to the results of the EMPEROR-Reduced study. The median survival time of the empagliflozin and standard treatment groups was 7.43 and 6.8 years, respectively.

The study showed that, compared with the placebo, the hazard ratio (HR) of the compound outcome for CV death or hospitalization for HF was 0.75 and the 95% confidence interval (CI) was 0.6–0.86 (*P* < 0.001); the HR for reduced hospitalization for HF outcomes was 0.69 and the 95% CI was 0.59–0.81 (*P* < 0.001). However, the outcome of CV death was not significant (HR = 0.92, 95% CI = 0.75–1.12, *P* < 0.001)

### Cost-effectiveness analysis

In this study, the Markov model was used to calculate the ICER and compare the WTP to determine the economic benefits of the intervention measures. The *China Guidelines for Pharmacoeconomic Evaluations 2020* recommend that one to three times the per capita GDP should be used as the ICER threshold; therefore, this study adopted one time the China’s per capita GDP ($11,981) in 2021 as the ICER threshold (23). The results of the cost-effective analysis are shown in Table 2. Compared with that of the standard treatment group, the ICER of the empagliflozin group was $3,842.20 per QALY, which is less than one time the China’s per capita GDP in 2021 ($11,981). Therefore, the use of the standard treatment regimen combined with empagliflozin for the treatment of HFrEF is more advantageous in terms of cost-effectiveness than the standard treatment regimen alone.

**TABLE 2:** The results by base-case analysis.

	Total cost ($)	Incremental cost ($)	Total life years (QALY)	Incremental life years (QALY)	ICER ($ pe QALY)
Empagliflozin group	6,112.53	1,040.02	5.89	0.27	3842.20
Standard treatment group	5,072.51		5.62		

ICER, incremental cost-effectiveness ratio; QALY, quality-adjusted life years.

### Sensitivity analysis

#### One-way sensitivity analysis

**FIGURE 2:**
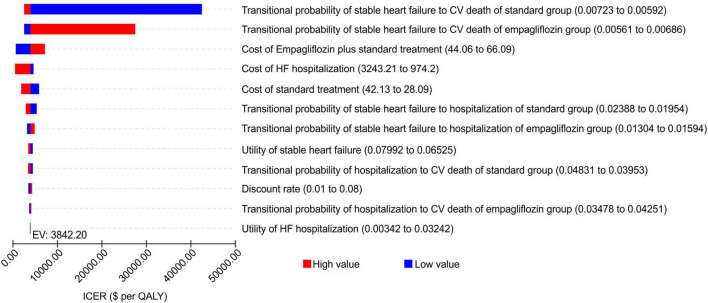
Tornado diagram showing the univariate sensitivity analysis of the Markov model simulation (empagliflozin group vs. standard group).

A one-way sensitivity analysis of the parameters of the Markov model showed that the reference was WTP [one time the per capita GDP ($11,981) of China in 2021]. The low value of the transitional probability of stable HF to CV death in the standard treatment group and high value of the transitional probability of stable HF to CV death in the empagliflozin group had a greater impact on the results, being more than one time the per capita GDP, and the steady-state mortality of the two groups had the greatest impact on the ICER. The other parameters had little influence on the ICER, and regardless of the changes in the range, the ICER remained below one time the per capita GDP of China in 2021. The tornado diagram of the one-way sensitivity analysis is shown in Figure 2.

#### Scenario analysis

By adjusting for the empagliflozin cost, cost of each hospitalization for HFrEF, and time horizon in the scenario analysis model, the results showed that when the empagliflozin cost decreased, the ICER decreased gradually; when the costs of hospitalization for HF in the different levels of hospitals increased, the ICER decreased gradually; and when the time horizon was extended, the ICER decreased gradually. This is shown in Table 3.

**TABLE 3:** The results by scenario analyses presented as the ICER.

Scenario	Value ($)	ICER ($ per QALY)
**Cost of empagliflozin**		
Descend by 10%	17.96	3,247.74
Descend by 20%	15.97	2,650.30
Descend by 40%	11.98	1,458.39
Descend by 60%	7.98	266.49
**Cost of different level hospitals**		
Town hospital	974.20	4,649.63
County hospital	1,132.53	4,355.02
Municipal hospital	1,804.13	3,105.35
Provincial hospital	1,853.54	3,013.41
Ministerial hospital	3,243.21	427.61
**Time horizon**		
10 years		5,223.66
15 years		4,237.51
20 years		3,842.20
25 years		3,695.45

ICER, incremental cost-effectiveness ratio; QALY, quality-adjusted life years.

#### Probability sensitivity analysis

**FIGURE 3:**
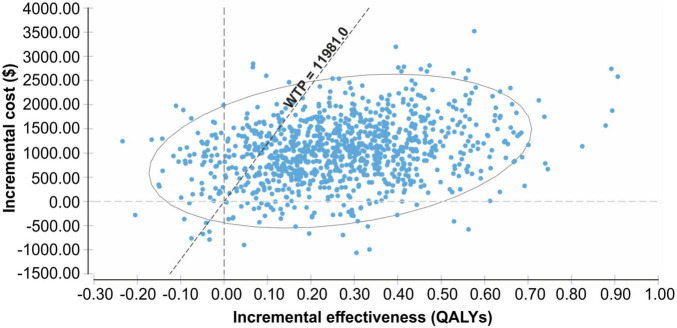
Scatter plot of the incremental costs and incremental quality-adjusted life-years from a thousand simulations for the empagliflozin group vs. standard group.

**FIGURE 4:**
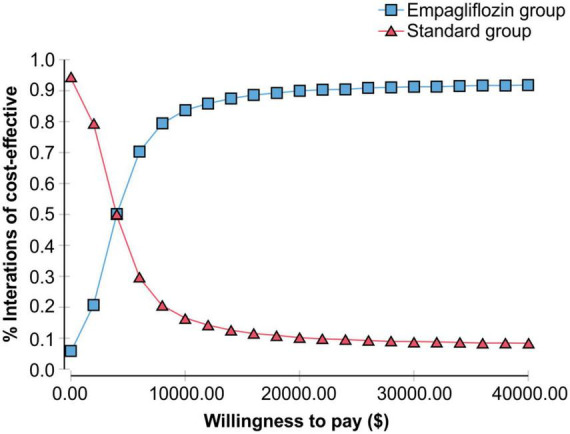
Cost-effectiveness acceptability curve showing the willingness to pay and the corresponding probability of cost-effectiveness for the empagliflozin group vs. standard group.

The probability sensitivity analysis of the Markov model was conducted using Monte Carlo simulation, and the ICER scatter plot diagram (Figure 3) and cost-effectiveness acceptability curve (Figure 4) were obtained. As shown in Figures 3, 4, when the WTP was one time the per capita GDP of China in 2021 ($11,981), the cost-effectiveness probability in the empagliflozin group was 85.8%; when the WTP was three times the per capita GDP of 2021 ($35.943), the cost-effectiveness probability of the empagliflozin group was 91.6%. When cost-effectiveness increased in the empagliflozin group, WTP increased.

## Discussion

In recent years, several studies have proven the benefits of SGLT2is for the treatment of HFrEF. Among them, EMPA-REG OUTCOME studies have shown that the treatment of patients with type 2 diabetes with confirmed CV diseases can reduce all-cause mortality and CV mortality (24). The DECLARE-TIMI 58 trial and CANVAS trial demonstrated that dapagliflozin and canagliflozin can improve the prognosis of patients with type 2 diabetes with CV diseases (25, 26). The results of the DAPA-HF study showed that dapagliflozin reduced the risk of CV death or hospitalization for patients with HF by 26%, and the risk of CV death was reduced by 18%; the results were consistent regardless of whether the patients had diabetes or not (27). The results of the EMPEROR-Reduced study showed that empagliflozin significantly improved the outcomes of patients with HFrEF, and its benefits on the major composite endpoints were mainly attributable to reducing the risk of hospitalization in patients with HFrEF (6, 7). The above-mentioned studies suggest that SGLT2i may improve the prognosis of HF; that is, the effects were similar. However, the EMPEROR-Reduced study did not show any benefit on CV death, which is the main difference between empagliflozin and dapagliflozin for the treatment of HFrEF.

The mechanism of the benefits of SGLT2is in HFrEF is the metabolic shift away from myocardial utilization of glucose (which is energy-inefficient) toward the consumption of free fatty acids and ketone bodies, which generate more ATP and enhance myocardial energetics (28). Enhanced myocardial energetics cause improvement in both systolic (29) and diastolic function (30). In humans, the use empagliflozin causes reverse left ventricular (LV) remodeling, with decrease in LV volumes and regression of LV hypertrophy (31), and a reduction in epicardial adipose tissue, aortic stiffness, and myocardial fibrosis (32). In addition, SGLT2is improve the quality of life in HFrEF (33).

Considering the possible widespread use of empagliflozin in patients with HFrEF in the future, decision makers should determine whether the additional benefits of empagliflozin are worth the extra cost. Liao et al. (34) conducted a pharmacoeconomic evaluation of the use of empagliflozin for the treatment of HFrEF in many countries or regions in the Asia-Pacific region, including Singapore, South Korea, Japan, Malaysia, Australia, Taiwan, and China. After adding empagliflozin, all countries and regions achieved better returns, with Singapore having the highest ICER value of $53,791 per QALY, followed by Japan ($24,046 per QALY), Australia ($20,982 per QALY), Taiwan ($20,508 per QALY), and South Korea ($8,846 per QALY). When the WTP was one and three times the local per capita GDP, the cost-effective probability was 58.1 and 94.2% in Singapore, 77.9 and 95.6% in Japan, 89 and 95.9% in Australia, 63.4 and 93.7% in Taiwan, and 93.6 and 96.3% in South Korea, respectively.

Based on the results of the EMPEROR-Reduced study and published literature, this study evaluated the economic effectiveness of empagliflozin for the treatment of HFrEF. Our findings show that the empagliflozin group has an ICER of $3,842.20 per QALY compared with that of the standard treatment group; that is, the cost of each additional QALY of the empagliflozin group was $3,842.20 more than that of the standard treatment group, which was lower than that of the WTP (one time the per capita GDP of China in 2021, $11981).

Based on the recommendations of the *World Health Organization* on pharmacoeconomic evaluation, increased cost was more economical when the ICER was less than one time the per capita GDP (22). This shows that the use of the standard regimen combined with empagliflozin for the treatment of HFrEF is more cost-effective than the standard treatment regimen alone.

From the results of the validation model, we assumed that the treatment of approximately 8.9 million Chinese patients with HFrEF with the standard regimen combined with empagliflozin will prevent approximately 1,005,700 hospitalizations for HFrEF and 89,000 deaths. The cost of hospitalization for HF will decrease by $1.408 billion, which will lighten the burden on China’s medical system.

The one-way sensitivity analysis showed that steady-state mortality was the most important factor affecting the ICER in both groups. This was similar to the results of the EMPEROR-Reduced study (9); that is, empagliflozin cannot reduce the CV mortality risk (HR = 0.92, 95% CI = 0.75–1.12) in patients with HFrEF. Therefore, changing the range of steady-state mortality in both groups will lead to significant changes in the ICER, which cannot be considered because of the instability of the model. As a result, when HFrEF cost decreased, the cost of hospitalization for HFrEF and treatment time increased. Hence, the addition of empagliflozin to the standard treatment regimen was more cost-effective. When WTP was $11,981 and $35,943, the cost-effective probability was 85.8 and 91.6% in the empagliflozin group, respectively, implying that the model was stable. The cost-effectiveness of the standard treatment regimen combined with empagliflozin was, thus, proven. Different countries have different health care systems and economic conditions. However, considering the results of the standard treatment regimen combined empagliflozin combined in the Asia-Pacific region mentioned above, we believe that the standard regimen combined with empagliflozin for the treatment of HFrEF is cost-effective in China, with the ICER relatively low and the economic probability relatively high.

This study had some limitations. First, the clinical data used in this study were obtained from the EMPEROR-Reduced study, which included a small number of Chinese patients, and the overall results deviate from the characteristics and outcomes of Chinese patients with HFrEF. Therefore, it is necessary to conduct a real-world study on the Chinese population in the future. Second, this study only considered direct medical cost and did not include direct non-medical, indirect, and hidden costs.

In conclusion, the standard regimen combined with empagliflozin for the treatment of HFrEF in Chinese patients was more cost-effective than the standard treatment regimen alone. This evidence-based information can guide clinical rational drug use and health decisions.

## Data availability statement

The original contributions presented in this study are included in the article/supplementary material, further inquiries can be directed to the corresponding author.

## Author contributions

HS: data curation, formal analysis, funding acquisition, methodology, project administration, resources, software, supervision, validation, and writing—review and editing. YW: data curation, formal analysis, investigation, methodology, software, and writing—original draft. ZM: data curation, formal analysis, and writing—original draft. SZ: data curation and writing—original draft and review and editing. QZ: investigation and writing—original draft and writing—review and editing. All authors contributed to the article and approved the submitted version.
